# Whole-Genome Sequencing Analysis of Salmonella Isolates from Poultry Farms, a Slaughterhouse, and Retail Stalls in Thailand

**DOI:** 10.1128/MRA.01063-20

**Published:** 2021-05-13

**Authors:** Kar Hui Ong, Kyaw Thu Aung, Sharon C. M. Chan, Swaine L. Chen, Lee Ching Ng, Kitiya Vongkamjan

**Affiliations:** a National Centre for Food Science, Singapore Food Agency, Singapore; b Environmental Health Institute, National Environment Agency, Singapore; c School of Chemical and Biomedical Engineering, College of Engineering, Nanyang Technological University, Singapore; d Nanyang Technological University Food Technology Centre, Nanyang Technological University, Singapore; e School of Biological Sciences, College of Science, Nanyang Technological University, Singapore; f Infectious Diseases Translational Research Programme, Department of Medicine, Yong Loo Lin School of Medicine, National University of Singapore, Singapore; g Laboratory of Bacterial Genomics, Genome Institute of Singapore, Agency for Science, Technology and Research, Singapore; h Department of Biotechnology, Faculty of Agro-Industry, Kasetsart University, Bangkok, Thailand; i Department of Food Technology, Faculty of Agro-Industry, Prince of Songkla University, Songkhla, Thailand; University of Maryland School of Medicine

## Abstract

The draft genome sequences of 21 Salmonella isolates obtained from poultry production chains in Hat Yai City, Songkhla Province, Thailand, are reported in this study. Our study revealed that there was Salmonella environmental contamination along poultry production chains and cross-contamination among poultry through inanimate surfaces in the environment.

## Abstract (precis)

The draft genome sequences of 21 Salmonella isolates obtained from poultry production chains in Hat Yai City, Songkhla Province, Thailand, were reported in this study. Our study revealed that there was Salmonella environmental contamination along poultry production chains and cross-contamination among poultry through inanimate surfaces in the environment.

## ANNOUNCEMENT

In Thailand, Salmonella is one of the common causes of foodborne infections (1), and poultry is the most consumed meat (2). Studies have shown that Salmonella contamination of poultry meat can occur during processing along the production chain (3). To understand the possible sources of Salmonella contamination, our study aimed to characterize Salmonella isolates from poultry production chains in Thailand using whole-genome sequencing analysis.

In 2016, 163 samples from Hat Yai City, Songkhla Province, Thailand, were collected—environmental samples from broiler farms (*n* = 32), environmental samples from a broiler slaughterhouse (*n* = 47), and environmental and raw chicken meat samples from wet market retail stalls (*n* = 84). A total of 56 Salmonella isolates were recovered from the samples using the ISO 6579:2007 protocol with slight modifications (4). Randomly selected Salmonella isolates (one colony per sample) were stored in brain heart infusion broth with 15% glycerol until further usage. Then, 21 isolates (from farms [*n* = 5], a slaughterhouse [*n* = 5], and retail stalls [*n* = 11]) were selected based on phenotypic resistance profiles and cultured in universal preenrichment broth at 37°C for 24 h, followed by DNA extraction using the DNeasy blood and tissue kit (Qiagen, Valencia, CA, USA). Library preparation was performed using the NEBNext Ultra DNA kit (New England Biolabs [NEB], USA). Samples were sequenced on a HiSeq 4000 sequencer (Illumina, San Diego, CA, USA) with 2 × 151-bp reads. All “pass filter” reads (as called using bcl2fastq version 2.20.0.422) were used in subsequent analyses. Multilocus sequence type (MLST) and resistance gene predictions were made using SRST2 version 0.2.0 (5) with MLST databases (6) from PubMLST (https://pubmlst.org) and the ARGannot resistance gene database (7). *De novo* assemblies were performed using Velvet version 1.2.10 with the VelvetOptimizer helper script version 2.2.4 (8) and a minimum contig cutoff of 500 bp, scaffolded with OPERA-LG version 2.0.6 (9), and finished with FinIS version 0.3 (10). The assembled genomes were annotated using Prokka version 1.13.3 (11) and analyzed with the following tools: *in silico* serotyping using SeqSero version 1.0 (12), identification of Salmonella pathogenicity islands (SPI) using SPIFinder version 1.0, and identification of plasmid replicons using PlasmidFinder version 2.1 (Centre for Genomic Epidemiology, Denmark) (13). Upon submission to GenBank, assemblies were reannotated using the NCBI Prokaryotic Genome Annotation Pipeline (14). Default parameters for software were used except where otherwise noted.

The draft genome sizes ranged from 4,661,649 to 5,100,460 bp with GC contents of 51.90 to 52.27% (Table 1). The number of contigs for each isolate ranged from 25 to 80. Sequence type 1541 (ST1541) (Salmonella enterica serovar Corvallis or Salmonella enterica serovar Chailey) was the most commonly predicted serotype (38.1%, 8/21). Out of 21 isolates, 16 (76.2%) were predicted to carry at least one resistance gene. Five types of plasmid replicons were found in 28.6% (6/21) of isolates. All isolates (except isolate SGEHI2016-PSU-BS-095SL) contained at least 3 types of SPIs. Our study suggested that Salmonella contamination had occurred in the environment along poultry production chains and that there was cross-contamination among poultry through environmental surfaces. Furthermore, our study provided baseline information on the genomic diversity of Salmonella isolates found in the poultry production chains in Thailand.

**TABLE 1:** Whole-genome sequencing characterization of 21 Salmonella isolates recovered from samples in farms, a slaughterhouse, and retail stalls in Hat Yai, Songkhla Province, Thailand

Sample ID^*a*^	Source	Sample description	Predicted serotype^*b*^	MLST^*c*^	Resistance gene(s)^*d*^	No. of resistance genes	Plasmid replicon(s)^*e*^	Salmonella pathogenicity islands^*f*^	Total no. of sequence reads	*N*_50_ length (bp)	No. of contigs	GC content (%)	Total length (bp)	Total sequence data (bp)	Genomic coverage (×)	SRA no.	GenBank accession no.
001SL	Farm	Cooling pad water	Weltevreden	365		0		SPI-2, SPI-3, SPI-4, SPI-8, C63PI	3,416,142	126,091	76	52.10	5,100,460	1,031,674,884	202	SRR12151691	JADDII000000000.1
010SL	Farm	Cooling pad water	Weltevreden	365	*aac3-IIa*, *aadA*, *inuF, bla*_TEM-1D_	4		SPI-2, SPI-3, SPI-4, SPI-8, C63PI	3,534,574	149,448	80	52.09	5,102,068	1,067,441,348	209	SRR12151690	JADDIH000000000.1
020SL	Farm	Fecal	Kentucky	314		0		SPI-2, SPI-3, SPI-4, SPI-8, C63PI	4,035,271	729,478	28	52.16	4,661,649	1,218,651,842	261	SRR12151679	JADDIG000000000.1
030SL	Farm	Feed	Potential monophasic variant of Typhimurium	34	*strA*, *strB*, *sulII*, *bla*_TEM-1D_, *tetB*	5	ColpVC	SPI-2, SPI-3, C63PI	3,641,947	278,606	53	52.15	4,958,996	1,099,867,994	222	SRR12151677	JADDIF000000000.1
037SL	Farm	Feed	Kentucky	314		0		SPI-2, SPI-3, SPI-4, SPI-8, C63PI	3,669,548	729,466	28	52.16	4,662,016	1,108,203,496	238	SRR12151676	JADDIE000000000.1
038SL	Retail stall	Cutting board swab	Corvallis or Chailey	1541	*strA*, *strB*, *sulII*, *tetA*	4		SPI-1, SPI-2, SPI-3, SPI-4, SPI-8, C63PI	3,951,543	793,388	30	51.91	4,881,101	1,193,365,986	244	SRR12151675	JADDID000000000.1
044SL	Retail stall	Cutting board swab	Corvallis or Chailey	1541	*qnr-S*, *strA*, *strB*, *sulII*, *tetA*	5		SPI-2, SPI-3, SPI-4, SPI-8, C63PI	4,137,978	793,383	32	51.91	4,890,864	1,249,669,356	256	SRR12151674	JADDIC000000000.1
052SL	Retail stall	Work table swab	Corvallis or Chailey	1541	*qnr-S*	1		SPI-1, SPI-2, SPI-3, SPI-4, SPI-8, C63PI	3,779,794	793,530	29	51.90	4,881,258	1,141,497,788	234	SRR12151673	JADDIB000000000.1
058SL	Retail stall	Work table swab	Kentucky	198	*aacAad*, *sulI*, *bla*_TEM-1D_	3	ColpVC, IncQ1	SPI-1, SPI-2, SPI-3, SPI-4, C63PI	3,454,419	817,359	31	52.21	4,880,320	1,043,234,538	214	SRR12151672	JADDIA000000000.1
067SL	Retail stall	Work table swab	Altona	1549		0	IncFII(S)	SPI-2, SPI-3, SPI-4, SPI-5, SPI-13, SPI-14, C63PI	3,903,860	547,503	25	52.27	4,688,587	1,178,965,720	251	SRR12151671	JADDHZ000000000.1
081SL	Retail stall	Cutting board swab	Corvallis or Chailey	1541	*qnr-S*, *strA*, *strB*, *sulII*	4	IncFII(S), Inc1-I(Gamma)	SPI-2, SPI-3, SPI-4, SPI-5, SPI-13, SPI-14, C63PI	3,791,130	412,818	33	51.91	4,890,404	1,144,921,260	234	SRR12151689	JADDHY000000000.1
082SL	Retail stall	Cutting board swab	Corvallis or Chailey	1541	*qnr-S*, *strA*, *strB*, *sulII*	4		SPI-1, SPI-3, SPI-4, C63PI	4,403,614	793,373	32	51.91	4,891,065	1,329,891,428	272	SRR12151688	JADDHX000000000.1
089SL	Retail stall	Carcass washing water	Corvallis or Chailey	1541	*qnr-S*, *strA*, *strB*, *sulII*	4		SPI-1, SPI-2, SPI-3, SPI-4, SPI-5, SPI-13, SPI-14, C63PI	3,779,688	521,418	36	51.91	4,892,070	1,141,465,776	233	SRR12151687	JADDHW000000000.1
095SL	Retail stall	Knife swab	Corvallis or Chailey	1541	*qnr-S*	1			3,435,933	521,294	40	51.91	4,877,861	1,037,651,766	213	SRR12151686	JADDHV000000000.1
099SL	Retail stall	Work table swab	Kentucky	198	*aacAad*, *sulI*, *bla*_TEM-1D_	3		SPI-1, SPI-3, SPI-4, C63PI	3,311,371	818,086	33	52.22	4,891,040	1,000,034,042	204	SRR12151685	JADDHU000000000.1
125SL	Retail stall	Chicken meat	Agona	13		0		SPI-1, SPI-3, SPI-8, C63PI	3,683,522	307,945	38	51.95	4,833,485	1,112,423,644	230	SRR12151684	JADDHT000000000.1
150SL	Slaughterhouse	Bucket swab	Kentucky	198	*aacAad*, *sulI*, *bla*_TEM-1D_, *tetA*	4		SPI-2, SPI-3, SPI-4, C63PI	3,814,780	773,805	33	52.20	4,800,040	1,152,063,560	240	SRR12151683	JADDHS000000000.1
154SL	Slaughterhouse	Feather separation swab	Singapore	462	*bla*_CMY_, *erm42*, *strA*, *strB*, *sulII, tetA*	6		SPI-1, SPI-3, SPI-4, SPI-5, SPI-13, SPI-14, C63PI	3,478,832	617,727	32	52.20	4,824,749	1,050,607,264	218	SRR12151682	JADDHR000000000.1
159SL	Slaughterhouse	Chicken pen swab	Kentucky	198	*aacAad*, *sulI*, *bla*_TEM-1D_, *tetA*	4		SPI-1, SPI-2, SPI-3, SPI-4, C63PI	3,844,880	773,582	30	52.20	4,799,895	1,161,153,760	242	SRR12151681	JADDHQ000000000.1
170SL	Slaughterhouse	Conveyor swab	Singapore	462	*bla*_CMY_, *erm42*, *strA*, *strB*, *sulII*, *tetA*	6	IncC	SPI-1, SPI-3, SPI-4, SPI-5, SPI-13, SPI-14, C63PI	4,116,456	466,641	33	52.20	4,824,348	1,243,169,712	258	SRR12151680	JADDHP000000000.1
179SL	Slaughterhouse	Wastewater	Corvallis or Chailey	1541	*qnr-S*	1	IncQ1	SPI-1, SPI-2, SPI-3, SPI-4, SPI-8, C63PI	3,944,301	743,296	28	51.96	4,845,458	1,191,178,902	246	SRR12151678	JADDHO000000000.1

a A prefix of “SGEHI2016-PSU-BS-” applies for all isolate IDs.

b Using SeqSero version 1.0.

c Using MLST databases from https://pubmlst.org/.

d Using the ARGannot resistance gene database supplied with SRST2.

e Using PlasmidFinder version 2.1 with a minimum identity of 95% and minimum coverage of 60%.

f Using SPIFinder version 1.0 with a threshold ID of 95% and minimum length of 60%.

### Data availability.

The raw reads and assembled genomes were deposited in GenBank under BioProject number PRJNA644105. The accession numbers for the individual isolates are listed in Table 1.
